# Nanoscale insights into hematology: super-resolved imaging on blood cell structure, function, and pathology

**DOI:** 10.1186/s12951-024-02605-2

**Published:** 2024-06-24

**Authors:** Jinghan Liu, Yuping Yolanda Tan, Wen Zheng, Yao Wang, Lining Arnold Ju, Qian Peter Su

**Affiliations:** 1 School of Biomedical Engineering, University of Technology Sydney, Sydney, NSW 2007 Australia; 2 School of Biomedical Engineering, The University of Sydney, Darlington, NSW 2008 Australia; 3 Charles Perkins Centre, The University of Sydney, Camperdown, NSW 2006 Australia; 4 Heart Research Institute, Newtown, NSW 2042 Australia

**Keywords:** Fluorescence nanoscopy, Super-resolution microscopy, Blood cells, Platelets, Red blood cells, White blood cells, Blood diseases

## Abstract

Fluorescence nanoscopy, also known as super-resolution microscopy, has transcended the conventional resolution barriers and enabled visualization of biological samples at nanometric resolutions. A series of super-resolution techniques have been developed and applied to investigate the molecular distribution, organization, and interactions in blood cells, as well as the underlying mechanisms of blood-cell-associated diseases. In this review, we provide an overview of various fluorescence nanoscopy technologies, outlining their current development stage and the challenges they are facing in terms of functionality and practicality. We specifically explore how these innovations have propelled forward the analysis of thrombocytes (platelets), erythrocytes (red blood cells) and leukocytes (white blood cells), shedding light on the nanoscale arrangement of subcellular components and molecular interactions. We spotlight novel biomarkers uncovered by fluorescence nanoscopy for disease diagnosis, such as thrombocytopathies, malignancies, and infectious diseases. Furthermore, we discuss the technological hurdles and chart out prospective avenues for future research directions. This review aims to underscore the significant contributions of fluorescence nanoscopy to the field of blood cell analysis and disease diagnosis, poised to revolutionize our approach to exploring, understanding, and managing disease at the molecular level.

**Graphical Abstract:**

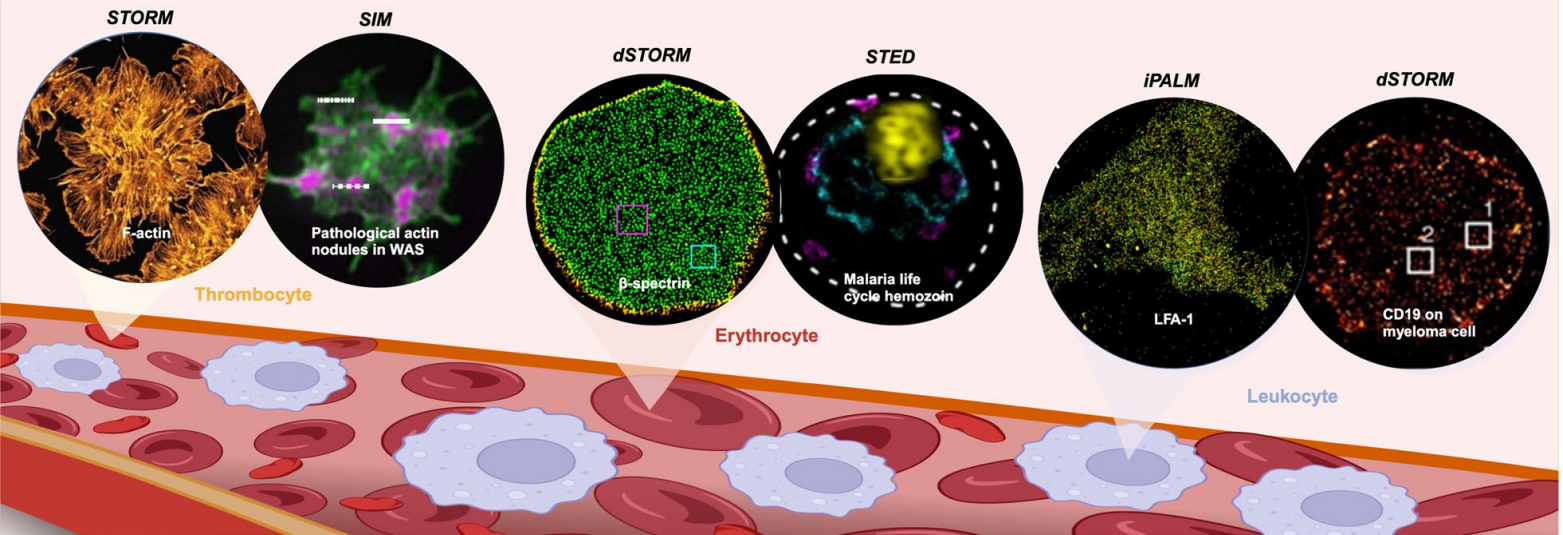

## Introduction

Blood cells, including erythrocytes (red blood cells), leukocytes (white blood cells), and thrombocytes (platelets), play crucial roles in maintaining homeostasis, defending against pathogens, and regulating thrombosis and haemostasis. Abnormalities in blood cell structure, function, or interactions can lead to various hematological disorders, such as anemia, thrombocytopenia, leukemia, and other bleeding or thrombotic disorders [1–4]. Understanding the nanoscale organization and dynamics of subcellular structures, proteins, and molecular interactions in blood cells is therefore fundamental to uncovering the mechanisms of blood cell function and dysfunction. “Seeing is believing”, conventional light microscopy techniques have been instrumental in studying blood cell morphology and functions, but the spatial resolution is limited [5]. The limitation is defined by two famous theories: Joseph J. Lister addressed a major resolution limitation in the earliest microscope designs in 1830 [6]; then Ernst Abbe established the famous diffraction limit formula in 1874 [7, 8]. The Abbe limit set the foundation for modern microscopy by establishing for the first time the theory of image formation and the diffraction limit of optical instruments, which states that the resolution of an optical microscope cannot exceed half of the detected light wavelength (about 200–300 nm for visible light wavelength) [5] as:

1 $$D = \frac{\lambda }{2n\sin \theta } = \frac{\lambda }{2NA}$$

where *D* is the smallest resolvable distance between two objects; *λ* is the wavelength of detection light; *n* is the refractive index of the optical system; *θ* is the angle of the cone of focused light in the objective; and *NA* is the numerical aperture of the objective.

This formula also guided the invention of advanced optical microscopy systems such as fluorescence confocal laser scanning microscopy (CLSM) and many others, which made optical microscopy a powerful and widely used platform for molecular and cell biologists. However, even with the optimized equipment and settings, light microscopy still cannot resolve structures smaller than 200–300 nm in lateral dimension (the x–y plane) and 500–700 nm in axial dimension (the x–z or y–z plane [5]). This is a challenge for studying tiny subcellular structures, especially in highly scattering tissues, where the optical slice thickness is restricted to 10–20 μm to maintain diffraction-limited resolution [9].

Many subcellular structures, including intracellular organelles and extracellular matrix (ECM) proteins, are small in their size and exhibit dynamic behaviors. Small organelles such as lysosomes and mitochondria, along with most proteins, are beyond the observational limits of conventional light microscopy [10–12]. The studies of some complex substances that exhibit high dynamics, including spliceosomes, nuclear pore complexes, DNA replication machinery, and the cytoskeleton, are also constrained by the time-consuming nature of conventional microscopy [13]. Fluorescence nanoscopy is highly demanded to overcome the restriction and reveal details of biological samples at the nanoscale, and some of them even with relatively high temporal resolution [14–17].

In recent years, the advent of super-resolution fluorescence microscopy techniques has revolutionized our ability to visualize biological structures at the nanoscale. These techniques, including structured illumination microscopy (SIM), stimulated emission depletion (STED) microscopy, and single-molecule localization microscopy (SMLM), among others, have overcome the diffraction limit and achieved spatial resolutions down to tens and even less than ten nanometers [18–20]. By enabling the visualization of previously unresolvable subcellular structures and molecular interactions, super-resolution microscopy has opened up new avenues for investigating blood cell biology and pathology.

Application of super-resolution microscopy to blood cells has already begun to yield novel insights. For example, SMLM has revealed the nanoscale organization of the actin-spectrin cytoskeleton in erythrocytes [21], while STED has uncovered the differential distribution of pro- and anti-angiogenic proteins in platelet granules [22]. However, many key questions remain unaddressed, such as: how do the nanoscale architectures of blood cells adapt to mechanical stress and biochemical signals? How do protein interactions and rearrangements at the nanoscale regulate blood cell function or dysfunction? What are the molecular signatures of hematological diseases at the nanoscale, and how can they be used for diagnosis or treatment monitoring? Answering these questions requires a systematic application of super-resolution microscopy to blood cell research.

In this review, we provide a comprehensive overview of the principles and applications of various super-resolution microscopy techniques in the context of blood cell imaging. We discuss the unique strengths and limitations of each technique, and highlight the key biological insights gained from their application to erythrocytes, leukocytes and thrombocytes. We further explore the potential of super-resolution microscopy for uncovering novel disease biomarkers and guiding therapeutic strategies. For example, understanding the nanoscale organization of the cytoskeleton in platelets may lead to the development of novel anti-platelet therapies for thrombotic disorders [23]. Similarly, elucidating the molecular mechanisms of parasite invasion and replication in erythrocytes using super-resolution microscopy could guide the development of targeted antimalarial drugs [24]. Finally, we discuss the current challenges and future directions in this rapidly evolving field and emphasize the need for multidisciplinary collaborations to fully harness the power of super-resolution microscopy in hematology research and clinical translation. We envision that super-resolution microscopy will become an indispensable tool in the diagnosis and treatment of hematological disorders, enabling personalized medicine approaches based on the nanoscale characterization of individual patients' blood cells.

## Super-resolution microscopy techniques

Fluorescence nanoscopy techniques effectively circumvent the diffraction limit theory, facilitating the visualization of subcellular structures and molecules at nanoscale resolution. The unprecedented resolution reveals insights that conventional optical microscopy cannot access. We summarize the current super-resolution technologies in this section, along with their optical capabilities, biological compatibility, practical boundedness, and technical limitations in Table 1.

**Table 1:** Fluorescence nanoscopy technologies: functionality and practicality

Nanoscopy	Technical principle	Spatial resolution	Live cell capacity	Sample prep	Illumination	Light dose	Technical limitations	Refs.
SIM/SSIM		XY: 100–150 nm Z: 200–300 nm	YES	Easy	Wide Field	Low–Medium	- Limited enhancement of resolution (approx. 2–3 folds) - High demand for data storage	[25–27]
STED		XY: 20–100 nm Z: 100–700 nm	YES	Medium	Scanning	High	- High laser power and special probes - Photobleaching/photo-damage - Trade-offs of resolution, imaging speed, and field of view	[28, 29]
PALM/(d)STORM SOFI/3B/DNA-PAINT		XY: 10–50 nm Z: 40–100 nm (iPALM Z: < 10 nm)	Hard	Easy–Medium	Wide Field	Medium–High	- Photo-activatable, photo-convertable, photo-switchable and/or photo-blinking probes - High laser power and long acquisition time - Photobleaching and toxicity - Complex imaging process steps	[14, 30–32]
ExM		XY: 40 nm Z: 170 nm	N/A	Difficult	Wide Field and/or Scanning	Low	- Complicated sample preparation - Incompatibility with some fluorescent probes or labels - Difficulties in expanding large, thick or dense samples	[33, 34]
MINFLUX		XYZ: < 5 nm	YES	Medium	Scanning	Low	- Complexity and high cost of the optical configuration - Labeling density and efficiency - Data analysis and validation	[35, 36]

Structural Illumination Microscopy (SIM), Saturated Structural Illumination Microscopy (SSIM), STimulated Emission Depletion (STED) Microscopy, Single-Molecule Localization Microscopy (SMLM), Photo-activated Localization Microscopy (PALM), (direct) Stochastic Optical Reconstruction Microscopy ((d)STORM), Super-resolution Optical Fluctuation Imaging (SOFI), Bayesian analysis of blinking and bleaching (3B), DNA Points Accumulation for Imaging in Nanoscale Topography (DNA-PAINT), Expansion Microscopy (ExM), Minimal Photon FLUXes (MINFLUX), Not Available (N/A)

### Structural illumination microscopy (SIM)

SIM utilizes wide-field illumination and creates laser gratings in different phase shifts, generating multiple fringe-like sinusoidal interference patterns that a mathematical model can reconstruct [25]. By acquiring images with the illumination pattern shifted to different positions and orientations, a high-resolution image can be computationally reconstructed, effectively doubling the resolution compared to conventional wide-field microscopy. It does not depend on fluorophores with specific photophysical or photochemical properties, allowing the use of any fluorescent probe to achieve a doubled X–Y resolution [5]. Consequently, SIM is less prone to photodamage and is one of the simplest super-resolution microscopies in terms of operation and sample preparation. With SIM, a lateral resolution of 100–150 nm, an axial resolution of 200–300 nm in three-dimensional (3D) and a temporal resolution of 1–100 ms can be achieved [26, 27].

The traditional SIM can only double the spatial resolution, as it is also limited by optical diffraction, similar to conventional microscopy [37]. By extending the SIM technique to Saturated Structured Illumination Microscopy (SSIM) and using photoswitchable probes, the resolution can be even future improved and is limited only by the signal-to-noise ratio [25]. The development of advanced reconstruction algorithms effectively enhances the resolution to ~ 60 nm [25, 38]. Wen et al. identified several limitations, including artifact production, computational cost and complexity, depth penetration, and contrast [39]. Novel algorithms are continuously being developed to address problems, such as High-fidelity structured SIM (HiFi-SIM), background Filtering SIM (BF-SIM) and many others [40].

This advanced method has the potential to provide higher resolution and contrast imaging of blood cells and platelets with lower artifacts and noise, and faster and simpler reconstruction, which could be groundbreaking for previous studies. In clinical settings, the improvements could serve as a diagnostic tool, aiding in the reduction of false positives or negatives in image analysis or diagnosis, and saving time and resources for imaging blood cells. Compared to other super-resolution techniques, SIM might be the best trade-off for spatial and temporal resolution. This will also make it easier for users to perform SIM reconstruction without extensive knowledge or expertise.

### STimulated emission depletion (STED) microscopy

STED effectively reduces the size of the point spread function (PSF) by selectively suppressing excited fluorophores in unselected regions using an annulus STED laser [41]. This concept can be likened to a swim ring or a doughnut, where the annular focus of the excitation laser emits fluorescent signals while the central intensity is set to zero. Compared to the other techniques, STED has an easier level of difficulty for sample preparation and operation procedure, and it is also a high-return nanoscopy technique that enhances the lateral resolution as 20–100 nm and the axial resolution as 100–700 nm.

While STED microscopy was first proposed in 1994, it was not applied to super-resolution imaging of blood cells and their associated proteins until 2012. The reason is STED faced many challenges and limitations in its early stages, such as high laser power that could cause photobleaching, photodamage, and heating of the sample, complex optics that required precise alignment and control of the doughnut-shaped beam of light, and limited availability and accessibility of the equipment and expertise [28]. STED microscopy has been developing for several decades and has achieved many improvements and breakthroughs in terms of resolution, contrast, speed, and versatility. A notable development in this field is REversible Saturable Optical Fluorescence Transition (RESOLFT) Microscopy, which uses a donut-shaped excitation beam to achieve high resolution while maintaining lower levels of residual fluorescence [42]. This approach is particularly promising in addressing the high laser power issue inherent in conventional STED nanoscopy.

Future enhancements could involve the integration of fluorophore replenishment strategies and the use of buffers to mitigate the effects of reactive oxygen species [29]. Additionally, advancing multicolor imaging, which is currently impeded by spectral crosstalk and chromatic aberration, remains a significant area for improvement [43].

### Single-molecule localization microscopy (SMLM)

The traditional molecular fluorescence labeling method has limited resolution due to the high degree of fluorophore overlap in biological samples. STED and SIM are both pattern excitation methods, with optical resolution represented by the effective size of the PSF. Stochastic Optical Reconstruction Microscopy (STORM and dSTORM) and Photo-activated Localization Microscopy (PALM) are the two main single-molecule positioning methods, with the position accuracy of a single probe as the main standard for measuring optical resolution [14, 15]. “One molecule at a time”, the new optical properties of fluorescent probes that can convert, switch and/or reverse between fluorescent and dark states have allowed for better spatial separation of diffraction-limited areas of molecular fluorescent probes [44]. SMLM has gained widespread use due to its relatively lower risk of photodamage, simpler sample preparation requirements, and its capability to achieve an impressively high resolution of ~ 20 nm laterally (~ 10 nm when a dual-objective setup is employed) and ~ 50 nm axially (~ 10 nm when interferometric PALM is introduced).

SMLM faces challenges that can affect the accuracy and reliability of reconstructed images, such as limitations in localization precision, fluorophore stability, and various sources of artifacts and labeling [30]. Despite these obstacles, notable advancements like the Super-resolution Optical Fluctuation Imaging (SOFI) [32], which utilizes fluctuating fluorescent markers, and DNA points accumulation for imaging in nanoscale topography (DNA-PAINT) [31, 45], employing DNA strands for precise targeting and unlimited ‘switching cycles’, have significantly improved resolution and enabled multicolor imaging in single-molecule studies. The innovations are part of a rapidly evolving landscape in SMLM, which holds great potential for future research.

SMLM enables multicolor, multiplexed, live-cell 3D imaging applications in blood cell studies [30]. STORM is a powerful technique for studying smaller subcellular structures in blood cells, it shows significant promise in analyzing spatiotemporal ultrastructural changes within small molecular proteins. So far, STORM is one of the most widely utilized super-resolution imaging tools for integrin investigations [30]. SMLM technique may contribute to novel therapeutic approaches in treating thrombosis and various other blood-related disorders by studying the key receptor, integrin αIIbβ3, involved in processes of platelet aggregation and activation [46, 47].

### Expansion microscopy (ExM)

ExM is a cost-effective technique that physically expands the sample using a swellable polymer network, allowing for improved imaging of fluorescent-labeled proteins similar to how a baby's diaper absorbs water [33]. However, this technique requires careful and superb sample preparation skills, including crosslinking, digestion, and expansion steps, to ensure uniform and isotropic expansion without compromising sample integrity or fluorescence [34]. Additionally, ExM cannot be performed with living cells and may not be suitable for certain large, thick or dense biological samples [48], for example, slices of the brain, embryos, and organs [49, 50].

ExM is particularly useful for studying integrin protein, as it preserves the molecular structure and interactions between integrin, the extracellular matrix, and the cytoskeleton, which are critical for its function [51]. The combination of dual-color expansion and confocal microscopy with colocalization analysis enables the quantification of highly dense receptors [52]. Nevertheless, ExM has some limitations, including the loss of approximately half of the fluorescent label due to physical expansion and the introduction of artifacts or distortions in the sample [53].

Despite these challenges, ExM is a promising technique that can achieve super-resolution imaging of the 3D structure and dynamics of integrin and other molecules in platelets and blood cells. Further optimization and validation are necessary to ensure the reliability and applicability of this technique for biological research.

### Minimal photon FLUXes (MINFLUX) microscopy

MINFLUX microscopy, a cutting-edge super-resolution microscopy technique, employs a donut-shaped excitation beam with a zero-intensity point to achieve precise and efficient localization of single molecules. Not only does MINFLUX minimize photodamage, but it also boasts the highest resolution among the discussed fluorescence nanoscopies. It is capable of reaching less than 5 nm resolution and can track fluorescent single molecules within a microsecond (μs) range [35].

While its capabilities are impressive, MINFLUX requires a specialized setup combining advanced optics, high-speed detection, and precise sample stabilization [35]. Implementing MINFLUX can be expensive and intricate, demanding high-quality components and specialized dyes [54]. Furthermore, the method demands meticulous labeling optimization, especially for living cells, and produces vast data sets that necessitate sophisticated, often custom, analysis tools [35, 36, 54]. Validating the bold resolution claims of MINFLUX also remains a hurdle, necessitating rigorous validation protocols [35].

From investigating molecular pathways to tracking the movements of membrane proteins, MINFLUX offers insights that were previously out of reach [35]. Such revelations promise to unlock deeper understandings of various cellular processes and could revolutionize diagnostics, therapeutics, and research in the field.

### Electron microscopy (EM)

EM has also contributed to the field by revealing lots of ultra-structures in blood cells, with the highest resolution (< 1 nm). On the other hand, EM has some disadvantages compared to light microscopy, such as expensive cost, unfriendly to living cells and complicated sample preparation [55]. For example, some of the latest EM techniques, such as scanning transmission electron microscope (STEM), focus ion beam-scanning electron microscopy (FIB-SEM) and serial block face-scanning electron microscopy (SBF-SEM), are not suitable for studying an ultra-structure, because the embedded sample is too thin to show the complete structure and protein content of the organelles [56]. This review focuses on light microscopy techniques and their applications, so we are not going to talk about EM in the following sections.

### Fixation reagents

It is worth noting that the fixation or crosslinking progress for fluorescence nanoscopy is critical to the imaging results and can cause artifacts [57]. 4% Paraformaldehyde (PFA) is the most widely used fixation reagent, usually with good staining, while has been found causing loss of antigenicity and changes in morphology. Glutaraldehyde (GA), compared with PFA, has a faster and stronger fixation with good morphology but with poor staining. Many research works utilized 4% PFA together with 1–2.5% GA to gain the optimized fixation. Glyoxal, sitting in the middle between GA and PFA, acts faster than PFA. Acetone and/or Methanol fixation can cause many artifacts with poor morphology. Choosing the right fixation reagents or combination should be practically optimized case by case.

## Deciphering subcellular architectures on blood cells

Fluorescence nanoscopy represents a cutting-edge technological advance in the field of single-molecule imaging, facilitating the detailed visualization of specific protein distributions and interactions within subcellular structures. It has been successfully applied to explore various subcellular structures, such as ultra-structure of erythrocytes, leukocyte membrane proteins interaction, and translocation. In this section, we summarize cytoskeleton, organelles and transmembrane protein distributions and organizations in different states platelets; membrane organization and clearance associated protein distribution in red blood cells; tetraspanin cluster interactions, membrane protein translocation and transmembrane protein organization in white blood cells **(**Table 2**)**.

**Table 2:** Ultra-architecture on blood cells revealed by super-resolution imaging techniques

Blood Cells	Technology	Structure and demonstrations		Figure	Refs.	Future research directions
Thrombocytes (platelets) Figure 1A	STORM	Proplatelet organelles distribution	Microtubules, mitochondria, dense-granule, actin, dense tubular system and α-granules	Figure 1B	[58]	*Cell membrane and membrane proteins* - Differences ratios of platelet integrin conformational states observation - Platelet integrin special subdomain (lamellipodium, filopodia, uropod, etc.) - Microtubules distribution during the formation of actin nodules on platelets - Lipid rafts with different conditions [59] and membrane deformation on red blood cells - Membrane organizers connections and interaction - Physical and chemical concepts in formation of tetraspanin nanodomain observation on leukocytes - Role of lipid raft boundaries in plasma membrane organization on leukocytes [60]
	STORM	Non-/activated-states organelles distribution	DTS, OCS, microtubules, actin nodules, actin bundles, mitochondria, autophagosomes, α-granules, dense granules, etc	Figures 1C/D	[56]	
	STORM	Fibrillogenesis related protein interaction	Fibronectin, fibrils, F-actin bundles and vinculin	Figure 1E	[62]	
	ExM	Transmembrane protein distribution	Overlapping fraction of αIIbβ3 related to platelet different activation state	Figure 1F	[63]	
	SIM	Platelet and megakaryocyte distribution	Actin, fibrinogen, microtubules	Figure 1G	[64]	
Erythrocytes (red blood cells, RBCs) Figure 2A	STORM	Membrane organization	Spectrin-actin-based cytoskeleton, junctional complex β-spectrin, F-actin, protein 4.1, tropomodulin, adducin, etc	Figure 2B	[21]	
	dSTORM	Clearance-associated proteins distribution	CD47, TSP1, SIRPα	Figure 2C	[65]	
Leukocytes (white blood cells, WBCs) Figure 2D	STED	Tetraspanin clusters distribution	CD53, CD37, CD81, CD82	Figure 2E	[66]	*Intracellular components (e.g. cytoskeleton)* - Proplatelet conversion into pre-platelets and the fission process study - Platelet actin filaments protrusion under flow - Real-time, live cell and multi-color imaging intermolecular process in activated platelet - Cytoskeleton deformable measurement under different in situ circulation conditions on red blood cells - Native red blood cells cytoskeleton observation exempt sample processing - Interaction between membrane-preserved cytoskeleton and actin filaments in red blood cells - PIP3 partitions positions, PIP2 and GM1 co-localization [61] on leukocytes
	SIM	Membrane protein translocation	F-actin between PMN and T-cell	Figure 2F	[67]	
	iPLAM	Transmembrane protein conformation measurement	Integrin αLβ2 (LFA-1, ICAM)	Figure 2G	[68]	

### Thrombocytes—platelets

**Fig. 1:**
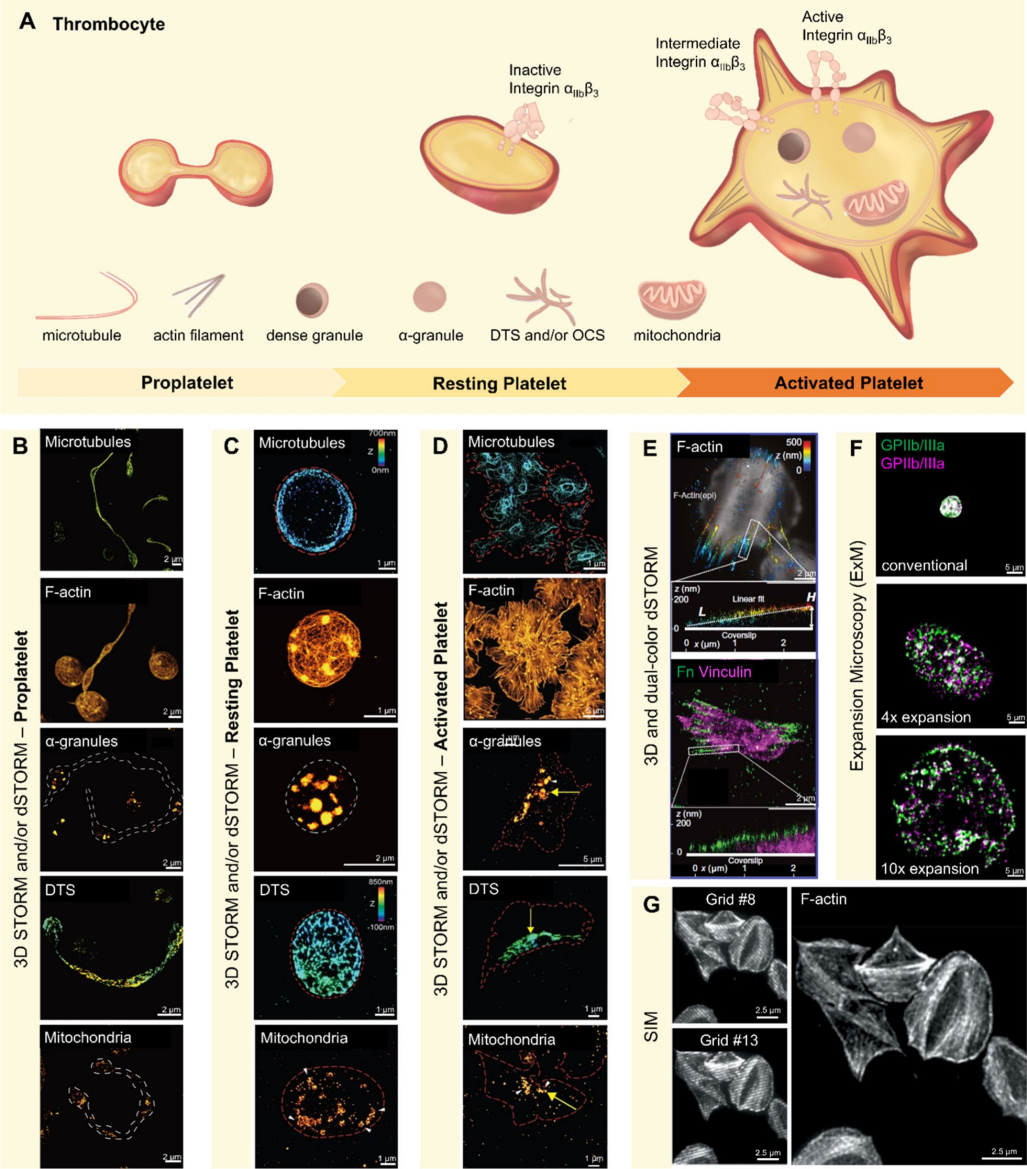
Fluorescence Nanoscopy Imaging Applications on Platelet Cells. **A** Illustration of different states of platelet and subcellular structures have been imaged by fluorescence nanoscopy. **B** Representative 2D/3D STORM images of multi-body proplatelets (one of the uncovered platelet intermediates), depicting cytoskeleton proteins and organelles, including immunolabeled microtubules (1st line); Alexa Fluor 647-conjugated phalloidin labelled actin filament (2nd line); anti-thrombospondin-1-fused thrombospondin-1 proteins on α-granules (3rd line); anti-KLC3-fused KLC3 proteins on DTS (line 4th); and anti-Tom20/Tom22-fused Tom20/Tom22 proteins on mitochondria (line 5th) [58]. **C** Representative 2D/3D STORM images describe distribution of cytoskeleton proteins and organelles in resting state platelets, including anti-α-tubulins-fused α-tubulins proteins on microtubules (1st line); phalloidin labelled actin filament (2nd line); nti-thrombospondin-1-fused thrombospondin-1 proteins on α-granules (3rd line); anti-KLC3-fused KLC3 proteins on DTS (4th line); and anti-Tom20/Tom22-fused Tom20/Tom22 proteins on mitochondria (5th line) [56]. **D** Representative 2D/3D STORM images describe the distribution of cytoskeleton proteins and organelles in activation state platelets with the same dye/antibodies chosen as above, such as acetylated microtubules in, after 20 min-activated platelets (1st line); phorbol 12-myristate 13-acetate (PMA) treatment actin bundles in, after 20 min-activated platelets (2nd line); the α-granules in PMA treatment-platelet activation 20 min (3rd line); the DTS in PMA treatment-platelet activation 20 min (4th line); the mitochondria in PMA treatment-platelet activation 20 min (5th line); yellow arrows demonstrate centralization of the organelles upon activation [56]. **E** Representative 3D STORM images of upward stretched platelets on fibronectin (fn) fibrils coated glass, including antibodies CF680-labelled F-actin, and its color-coded z position from blue to red, which is basal to apical (1st line); representative dual-color 3D STORM images of upward stretched platelets on fibronectin (fn) fibrils coated glass, including antibodies pFn647 labelled fn in green and anti-vinculin labelled vinculin in magenta (2nd line). Both dash line expanded images are side views of length L and the height H of a single Fn fibril (top panel) [62]. **F** Representative ExM images of resting platelets with different levels of expansion, including Alexa Fluor 488 (green) and Alexa Fluor 594 (magenta) labelled αIIbβ3 in an original platelet (1st line); Alexa Fluor 488 (green) and Alexa Fluor 594 (magenta) labelled αIIbβ3 in 4-times-expanded platelet (2nd line), and Alexa Fluor 488 (green) and Alexa Fluor 594 (magenta) labelled αIIbβ3 in a 10-times-expanded platelet (3rd line) [63]. **G** Representative raw SIM images (left, #8 image and #13 image from total of 15 raw images where a grid is iteratively rotated) and final SIM image (right) of phalloidin labeled F-actin in platelets spread on fibrinogen [64]

Fluorescence nanoscopy has opened new vistas for visualizing the subcellular architecture of platelets, offering insights into their maturation, resting state, and activation. These advanced imaging modalities have become indispensable tools in unraveling the complexities of platelet biology, from their genesis in the bone marrow to their critical role in hemostasis and thrombosis. Super-resolution microscopy provides unprecedented opportunities to observe key subcellular structures, such as intracellular organelles, membrane proteins, and cytoskeletons, and track their distributional changes during platelet maturation and activation processes, induced by biochemical and biomechanical stimuli [69]. By visualizing the orchestration of membrane receptors, organelles, and cytoskeletal components, these techniques highlight the profound structural transitions accompanying platelet activation, offering molecular insights crucial for exploring the hemostatic process, thrombotic disease progression, and the enhancement of existing anti-platelet therapeutic approaches (Fig. 1A).

#### Platelet maturation from megakaryocytes to proplatelets

At the forefront of platelet genesis, STORM imaging by Doory Kim and colleagues [58] has provided a window into the transformation of megakaryocytes into proplatelets, uncovering the ultrastructural organization of organelles during different intermediate stages of platelet maturation (Fig. 1B). This nanoscopic technique documents the dynamic rearrangement of cytoskeletal elements, microtubules and actin filaments, and their crucial roles in shaping the platelet and positioning other organelles like mitochondria, dense granules, and α-granules, through the various stages of platelet maturation.

#### Platelet in resting states: structural details

The resting state of platelets features an elaborate arrangement of receptors, exemplified by dense clusters of the adhesion receptor integrin αIIbβ3 (also known as CD41 and GPIIb/IIIa), a major platelet adhesion receptor and crucial for platelet aggregation and subsequent formation of hemostatic plug or pathological thrombus [47, 70]. Expansion Microscopy (ExM) has been pivotal in exploring these complexes, providing detailed visualizations of these protein organizations in resting and activated platelets alike [63, 71]. By achieving two expansion levels (4X and 10X), ExM can better visualize high-density αIIbβ3 clusters in both resting and activated platelets (Fig. 1F). ExM's ability to expand biological samples has proven to be a game-changer, offering a high-resolution perspective into the spatial distribution of αIIbβ3 receptors primed for activation.

#### Platelets in activation states: aggregation and thrombosis

The transition to platelet activation, a critical step towards thrombus formation, is characterized by significant structural changes at the nanoscale, as delineated by the combined efforts of STORM and electron microscopy in the studies by Kim et al. [56]. These techniques have precisely captured the dynamics of actin and microtubule bundles, shedding light on their essential roles in granule fusion and the release of cargo via the open canalicular system (OCS, Fig. 1C and D).

Complementary to these insights, 3D STORM has been employed by Lickert et al. [62] to scrutinize the nanoscale interaction of platelets with the extracellular matrix, revealing the differential assembly of plasma fibronectin architectures (Fig. 1E). In parallel, SIM has been used to reveal the detailed distribution of fibrinogen, a key ligand for αIIbβ3, across the platelet surface, offering a marker for different activation states [64] (Fig. 1G). This level of detail enriches our understanding of the molecular choreography that dictates platelet activation and the development of thrombi, paving the way for novel therapeutic interventions.

### Erythrocytes—red blood cells

#### Membrane cytoskeletal proteins

**Fig. 2:**
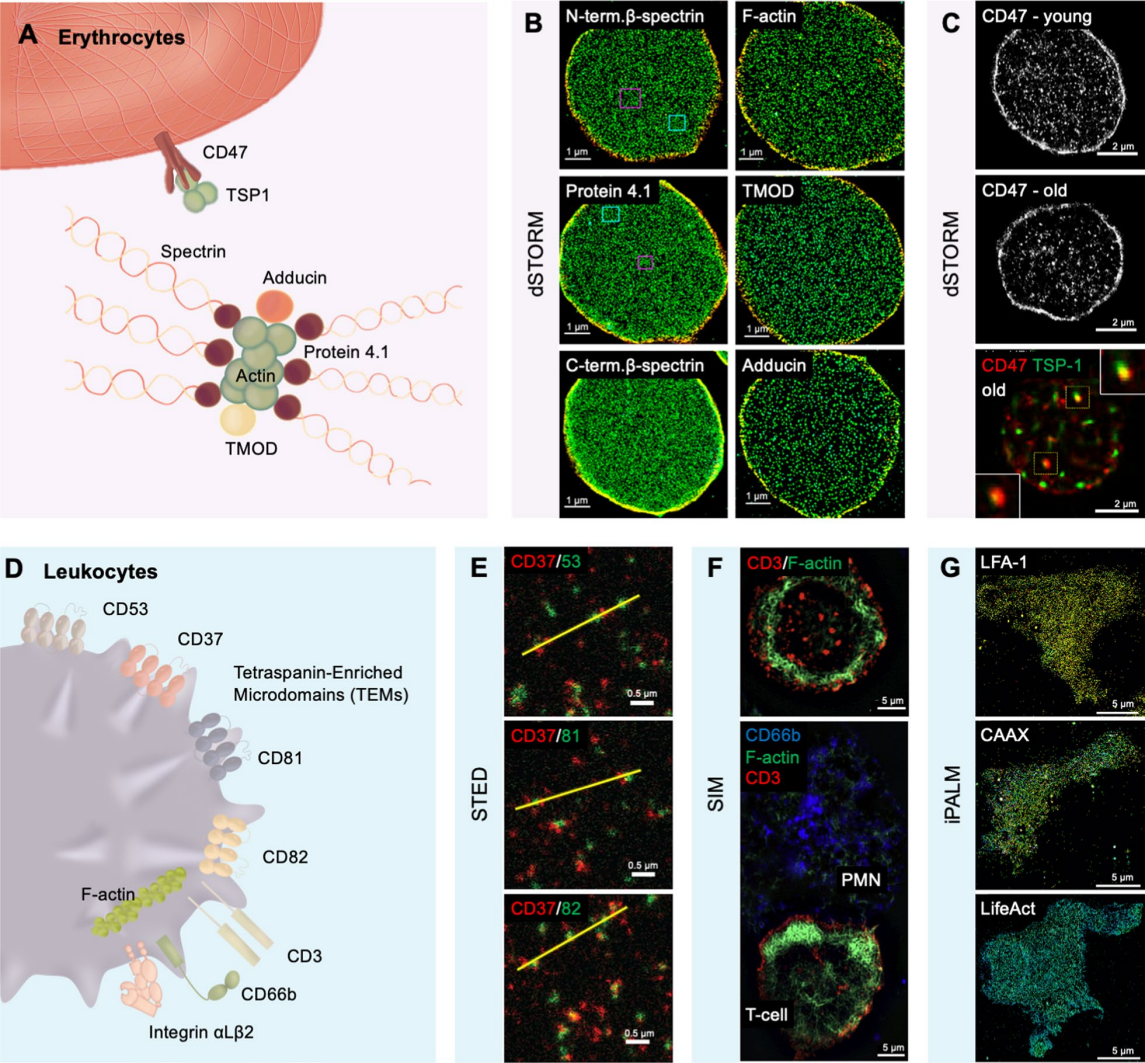
Fluorescence Nanoscopy Imaging Applications on Red Blood Cells and White Blood Cells. **A** Illustration of red blood cells’ membrane organization and protein distribution have been imaged by fluorescence nanoscopy. **B** Representative 3D STORM images of the cytoskeleton proteins of membrane-preserved RBCs, including antibodies ABT185 labelled N-termini (actin-binding domain) of β-spectrin and phalloidin labelled of actin filaments (1st line); antibodies protein 4.1R labelled protein 4.1 and antibodies TA503146 labelled Tropomodulin 1 (TMOD) (2nd line); antibodies 73–374 labelled C termini of β-spectrin and ab51130 labelled adducin (3rd line) [21]. **C** Representative dSTORM images of cd47 + / + mouse RBCs, including anti-CD47 fused AF647 on CD47 in young sample (1st line) and CD47 in aged sample (2nd line); and dual‐color STORM co‐localization images of TSP‐1-fused TAMRA on TSP‐1 and anti-CD47 fused AF647 on CD47 (3rd line) [65]. **D** Illustration of white blood cells’ membrane protein organization and interaction have been imaged by fluorescence nanoscopy. **E** Representative dual-color STED images of tetraspanin cluster’s proteins on B cell membrane, including antibodies WR-17 labelled CD37 in red and CD53(Mo) fused Ig-KK114 labelled CD53 in green (1st line); antibodies WR-17 labelled CD37 in red and antibodies JS-81 labelled CD81 in green (2nd line); antibodies WR-17 labelled CD37 in red and antibodies B-L2 fused AHP1709 labelled CD82 in green (3rd line) [66]; **F** representative SIM images of translocation proteins of PMN/T-cell conjugate, including T cell’s PeTxR labelled CD3 in red and SiR labelled actin in green (1st line); above and additionally mark PMN cell’s through FITC labelled CD66b in green (line 2nd) [67]. **G** Representative iPALM images of Jurkat cells with color-coded z position from red to bule (basal to apical) migrating on ICAM-1, which expresses mEOS3.2 labelled LFA-1(1st line); mEOS3.2 labelled CAAX (line 2nd); LifeAct-fused mEos3.2 labelled actin (3rd line) [68]

The erythrocyte cytoskeleton has been understood to consist of spectrin tetramers, which are joined to form a triangular lattice at the junctional complex that is composed of molecules such as actin filaments, adducin, tropomodulin and protein 4.1 [21] (Fig. 2A). By using STORM imaging, Pan and colleagues [21] revealed the existence of nanoscale gaps within the cytoskeleton with higher resolution and provided a more detailed and comprehensive nanoscale cytoskeletal structure, which has not previously been observed with conventional microscopy techniques (Fig. 2B). They also showed that the erythrocyte cytoskeleton has a heterogeneous and dynamic organization that may have implications for its functions and interactions with membrane proteins.

#### CD47-TSP-1 interactions

CD47 on the erythrocyte membrane surface, together with TSP-1, are thought to be involved in the clearance of aged erythrocytes [65]. dSTORM allowed for the observation of CD47 and TSP-1 at the nanoscale level, Wang et al. [65] revealed significantly different distribution patterns between young and aged erythrocytes. Specifically, aged erythrocytes had a decreased density of CD47 clusters but an increased CD47-TSP-1 interaction via a lipid raft-dependent mechanism that further enlarges the size of CD47 clusters (Fig. 2C). The study also demonstrated that the binding affinity of CD47 and TSP-1 is determined by the spatial distribution of the clusters rather than the total number of molecules. The nanoscale structure provided direct evidence for the role of TSP-1 in signaling the clearance of aged erythrocytes.

### Leukocytes—white blood cells

Building upon the insights provided by super-resolution nanoscopy, this sophisticated imaging extends into the realm of leukocyte biology, revealing the intricate subcellular structures and interactions that orchestrate immune responses. These techniques have shed light on the organization of membrane proteins, such as tetraspanins, and the dynamics of integrins, which are critical for leukocyte function and intercellular communication (Fig. 2D).

#### Tetraspanin-enriched microdomains (TEMs)

Nanoscopy has provided a detailed visualization of TEMs within the leukocyte plasma membrane, which serve to organize and compartmentalize cellular processes. Through STED microscopy, Zuidscherwoude et al. [66] unveiled the intricate organization of these domains, demonstrating that CD37, CD53, CD81, and CD82 form adjacent, non-overlapping nanoclusters, a structural configuration essential for B cell function (Fig. 2E).

#### Polymorphonuclear neutrophils (PMN) and T-cell interactions

The interactions between PMNs and T cells are crucial for the innate and adaptive immune responses. Advanced nanoscopy has enabled the observation of these cellular interactions in unprecedented detail. Balta et al. [67] utilized SIM in conjunction with InFlow microscopy to visualize the dynamic contacts between these cells in a flowing environment, highlighting the associated translocation of adhesion molecules such as CD66b and CD3 along with F-actin filaments (Fig. 2F). These findings provide new insights into the mechanisms and functions of PMN/T-cell interactions in health and disease.

#### Leukocyte integrin dynamics

In lymphocytes, on type of leukocytes, integrins like αLβ2 (also known as lymphocyte function-associated antigen 1, LFA-1) are pivotal for a myriad of cellular processes, including adhesion and migration [72]. Employing techniques such as SMLM, researchers have delved into the dynamic behavior of these proteins. For instance, Moore et al. [68] utilized iPALM to detail the displacement of LFA-1 integrins by measuring the precise distance of integrins with different conformations. They found that the head of integrin LFA-1 aligned with the actin reverse flow direction (Fig. 2G). Highlighting this, a landmark study in 2010 [73] leveraged interferometric Photo Activated Localization Microscopy (iPALM) to intricately map the nanoscale architecture of integrin-based cell adhesions, an area of research relevant beyond the specific study of blood cells. This important discovery highlights the potential of iPALM microscopy for measuring integrin conformational changes and mapping its related construction at nanoscale resolution (< 10 nm in Z axis).

#### Exploring leukocyte surface interactions

Super-resolution imaging has also illuminated the transient nanoscale reorganization of surface proteins critical for leukocyte function, such as CD44 clustering during cell rolling over E-selectin, which is a key step in the leukocyte extravasation process [74]. This level of detailed visualization offers new opportunities to understand the molecular basis of leukocyte behavior and interactions in the context of immune surveillance and disease.

As super-resolution nanoscopy continues to reveal the complexities of leukocyte molecular architecture, these techniques not only complement our understanding of leukocyte biology but also open up new avenues for diagnosing and treating immune-related diseases. For example, PMN and T cell interactions can be used as biomarkers for diagnosis or prognosis of inflammatory and autoimmune diseases, such as rheumatoid arthritis, multiple sclerosis, inflammatory bowel disease, or sepsis [75–79].

## Exploring potential biomarkers at the molecular level

Fluorescence nanoscopy transcends the limits of conventional imaging techniques and unveils the subcellular structures with high resolution, which has opened new horizons in identifying and understanding potential biomarkers based on deciphering the subtle nuances of cellular distributions in blood cells. As we delve into the microscopic world with unprecedented clarity, potential biomarkers for the diseases related to blood cells, such as integrin αIIbβ3 and CD63 on platelets, CD19 on myeloma cell, are revealed by super resolution microscopy. Fluorescence nanoscopy not only challenges our existing knowledge but also paves the way for innovative therapeutic validation and diagnosis, thereby contributing to the advancement of molecular biology and medical diagnostics (Table 3).

**Table 3:** Super-Resolution Imaging for Blood Cell Associated Disease

Diseases	Nanoscopy	Demonstrations (molecules/organelles)	Specificity/sensitivity	Figure	Refs.
**Rare platelet disorder**					
Type II Glanzmann Thrombasthenia (GT)	dSTORM	Distribution of cytoskeleton, F-actin, vinculin, myosin IIA, α-actinin, actin fibers, vinculin adhesion sites	Platelet isotropic phenotype	Figure 3A	[80]
Wiskott–Aldrich Syndrome	SIM and STORM	Organizations of actin nodules Interaction of actin cytoskeleton and ECM	Platelet actin nodules absence	Figure 3B	[81]
Hermansky–Pudlak Syndrome	SIM	Distribution of CD63 granules	Platelet CD63-positive phenotype	Figure 3C	[82]
**Cancer**					
Cancer, cardiovascular disease, bleeding disorder	STED	Distribution of pro-angiogenic VEGF, anti-angiogenic PF-4, fibrinogen (Fg), actin	VEGF, PF-4, Fg: differences in sizes and numbers Actin: thrombin induces filopodia/ADP induces monomer form	Figure 3D	[22, 43]
Glioma, cervical endometrial and ovarian cancers	SIM	Distribution of α-granules, microtubules, dense granules from tumor /benign mass patients & healthy volunteers	Double the number of α-granules in platelet	Figure 3E	[83]
Ovarian cancer	STED	Interaction of cancer progression and metastasis-related protein (P-selectin, integrins, mucin-binding receptors)	Most of P-selectin re-distribute in circular	Figure 3F	[84]
Breast cancer, ovarian cancer	STED	Interaction of SNAREs & angiogenesis-regulating proteins in platelet-mediated cancer progression	Classification models built	Figure 3G	[85]
Myeloma	dSTORM	Distribution of CD19, CD20 on myeloma cells	With < 100 antigens per myeloma cell	Figure 3H	[86]
**Infectious diseases**					
Plasmodium	STED	Distribution of Plasmodium spp. parasites, micronemes & vacuole of trophozoites	Various life cycle stages	Figure 3I	[87]
Covid-19 (SARS-CoV2)	dSTORM	Interaction of PF4 and vaccine components forming antigenic	Immunogenic complexes formation	N/A	[88]

### Rare platelet disorders

Platelet disorders refer to abnormalities in platelet numbers and functions, either too many (thrombocythemia, thrombocytosis) or too few (thrombocytopenia), or in their functional state (dysfunction) [89]. These abnormalities can lead to bleeding or hemostatic disorders. Some rare platelet disorders, such as Type II Glanzmann Thrombasthenia (GT) and Hermansky-Pudlak Syndrome (HPS), are challenging to recognize and diagnose [80, 82]. Fluorescence nanoscopy, as an advanced observational tool, has already developed novel biomarkers for some rare platelet disorders and holds the potential to be part of an effective platform for diagnosis and prognosis **(**Table 3**)**.

#### Type II glanzmann thrombasthenia (GT)

**Fig. 3:**
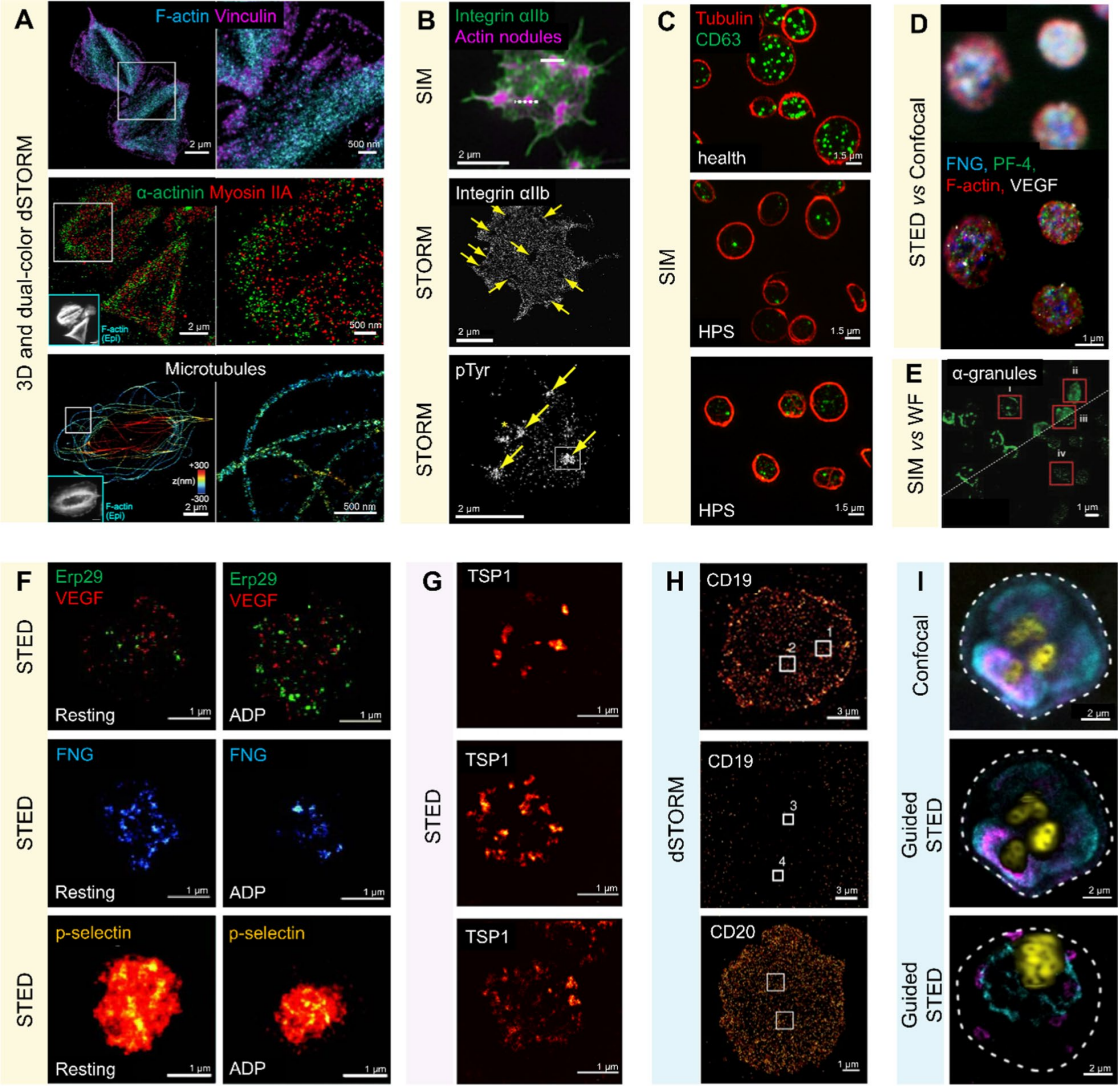
Fluorescence Nanoscopy Imaging Applications for Blood Cell Associated Disease. **A** Representative dual-color dSTORM images, describes the distribution of cytoskeleton proteins and organelles on the left and right (zooming pixel), including the phalloidin-AF488 labeled actin in cyan and anti-vinculin labeled vinculin in magenta (1st line); antibodies A5044 labeled α-actinin in green and anti-myosin IIa labeled myosin IIA in red (2nd line); Representative 3D dSTORM anti-tubulin labelled microtubules from red to blue (basal to apical) (3rd line). All the inset epifluorescence images are F-actin in the same cells [80]; **B** Representative 3D SIM image of human platelet proteins, Alexa568-phalloidin labelled in magenta and FITC-anti-αIIb labelled αIIbβ3 in green, line scans indicate actin intensity (1st line); Representative dSTORM TIRF image of human platelet proteins, including Alexa488-phalloidin labelled F-actin, the arrows indicate actin nodules in the corresponding integrin depleted zones (2nd line); and Alex647-anti-pTyr labelled tyrosine phosphorylated proteins (pTyr), the asterisk indicates an actin nodule that resolves as two separate foci of pTyr and the arrows indicate actin nodules in the corresponding integrin depleted zones (3rd line) [81]. **C** Representative SIM images of CD63-positive granules in platelets from a healthy control (1st line, distinctly and strongly colored) and two HPS patients (2-3rd line, less intense), anti-CD63 labelled CD63 in green and anti-tubulin labelled tubulin in red [82]. **D** Representative confocal (1st line) and four-colored STED (2nd line) images of proteins in platelet, including Alexa594-phalloidin labelled actin in red, Alexa594-ab labelled fibrinogen in blue, ATTO647N-ab labelled PF-4 in green, and Dylight650-ab labelled VEGF [43]. **E** Representative comparison joint images of wide-field (WF) and SIM of anti-human von willebrand factor fused VWF proteins on α-granules in tumor patient’s platelet [83]. **F** Representative STED images of resting and ADP-activated platelet in left and right, anti-Erp29 labelled Erp29 in green and anti-VEGF labelled VEGF in red (1st line); anti- fibrinogen labelled fibrinogen, FNG (2nd line); anti-P-selectin labelled P-selectin (3rd line) [84]. **G** Representative STED images of platelets with anti-tsp-1 labelled thrombospondin-1 (TSP-1) under different co-culturing conditions, including resting control (1st line); co-cultured with cell line MCF-10A (2nd line); co-cultured tumor cell lines EFO-21 (3rd line) [85]. **H** Representative dSTORM images of the detection of anti-CD19 labelled CD19 in a CD19-positive myeloma cell (1st line); and anti-CD19 labelled CD19 in a CD19-negetaive myeloma cell (2nd line); anti-CD20 labelled CD20 in a CD20-positive myeloma cell (3rd line) [86]. **I** Representative confocal (1st linn) and guided-STED images of malaria life cycle hemozoin in RBCs, including anti-EXP2 labelled EXP2 (the core component of the parasite protein translocon) in cyan and anti-ERC labelled parasite endoplasmic reticulum in magenta (2nd line); anti-EXP2 labelled EXP2 in cyan and SBP1 labelled Maurer’s clefts in magenta (3rd line) [87]

Lickert and his teams [80], by utilizing dSTORM, visualized a distinctive morphological fingerprint in the form of a bipolar phenotype on single platelets from healthy donors. However, platelets from a patient with Type II GT exhibited an isotropic phenotype due to αIIbβ3 deficiency (Fig. 3A). Hence, the bipolar phenotype is characterized as a morphology indicative of integrin αIIbβ3 outside-in signaling. This distinct isotropic phenotype in platelets may act as a biomarker for thrombocytopenia. Moreover, its integration with Lickert's team's morphometric screening method offers new opportunities for classifying elusive bleeding disorders. Additionally, their research sheds light on why α5β1 replaces αIIbβ3 to form fibronectin fibrils in Type II GT, a finding that could be crucial in developing novel therapeutic strategies.

#### Wiskott–Aldrich syndrome (WAS)

WAS is a rare genetic disorder that causes thrombocytopenia and bleeding disorders as well. Actin nodules and filaments, regulated by the activity of Wiskott–Aldrich Syndrome Protein (WASp) and the Human Actin-Related Protein 2/3 Complex (Arp2/3), play a critical role in platelet activation. Based on this, Poulter and his team [81] conducted advanced microscopy on exploring organization construction and signaling pathways of actin nodules (Fig. 3B) and uncovered the pathogeny of WASp at the molecular level. Their study, which utilized both SIM and dSTORM techniques, highlights the advantages of employing multiple types of nanoscopy techniques at the molecular level research, enhancing robustness and efficiency. Besides, the isoforms of Arp2/3 complex, Arp2/3 complex subunit 1B (ARPC1B), have been reported related to inflammatory diseases, and ARPC1B deficiency proplatelet shows significant differences under SIM imaging [90].

#### Hermansky–Pudlak Syndrome (HPS)

Clinical research revealed that 76% of HPS patients were initially misdiagnosed, and 28% had to consult with four to six doctors before receiving a correct diagnosis [82]. Thus, the diagnosis of platelet granule disorders (PGDs) necessitates a diagnostic assay that ensures accuracy while being both fast and efficient. [91]. According to the advantages mentioned above, SIM it is the most frequently used tool in clinical as a disease diagnosis tool, especially in platelet granules-related diseases. Cutler and his team employed SIM to find a novel biomarker of HPS, a CD63-positive distribution in platelet-dense granules [82]. The patients with HPS had fewer CD63-positive granules, which displayed weak staining, and there appeared to be more CD63 on the platelet periphery (Fig. 3C). The method they proposed combined with an automated image analysis workflow will bring a novel diagnosis clinically with speed and accuracy.

### Cancer

The failure to timely detect and diagnose cancer contributes significantly to high mortality rates [92]. Therefore, identifying novel cancer biomarkers is crucial for early diagnosis and effective treatment in later stages. Fluorescence nanoscopy, as a high resolution, fast response, and deep imaging tool, has significantly facilitated the identification of valuable biomarkers for oncological diagnosis and therapeutic monitoring. While the importance of extracellular vesicles (EVs) as sources of biomarkers for cancer diagnosis is well recognized [93], recent emphasis has expanded to include the examination of various biochemical changes at the subcellular level (such as reactive oxygen species, pH alterations, enzymes) [94]. However, in this section, we will specifically focus on the subcellular level cancer diagnosis and monitoring, examining distribution, interaction and organization of cancer-related subcellular structures in platelets and myeloma cells.

Mounting evidence suggests that thrombocytosis is linked with an increased risk of both the development and mortality of various cancers [95]. Platelet count may serve as a crucial biomarker in cancer screening and prognosis, which underscores the significant role of platelets in carcinogenesis and metastasis [95]. Platelets aid in tumor progression and metastasis not solely through physical interactions with cancer cells but also through functional engagements. They primarily respond to signals emitted by tumor cells, including changes in coagulation parameters [96]. Intriguingly, research has found that even a tiny tumor size (< 1 mm) relies on the development of new blood vessels for growth [97]. Therefore, platelets are not only associated with bleeding disorders and thrombosis but also with the severity of tumorigenesis and tumor metastasis.

#### Alpha-granules patterns

Recent studies have revealed distinctive nanoscale alterations in the distribution and organization of platelet proteins in cancer patients compared to healthy individuals. Zhang et al. [83] utilized SIM to identify circular coiling patterns of α-granules and microtubules in platelets from patients with glioma, cervical, endometrial, and ovarian cancers (Fig. 3E). These unique structural arrangements were not observed in healthy controls, suggesting their potential as novel diagnostic biomarkers. Notably, this research integrates fluorescence nanoscopy with deep learning to analyze a large dataset of platelet super-resolution images, with a sample size of 280,000, demonstrating that AI could be an effective tool for nanoscopy-based clinical diagnosis in the future. Mechanistically, the altered α-granule organization may reflect aberrant platelet activation in the prothrombotic state associated with cancer, which is known to promote metastasis [98].

#### Angiogenesis-regulating proteins

STED imaging has further revealed differential distribution of pro- and anti-angiogenic proteins like VEGF and PF-4 in platelets from cancer patients [22, 43]. Rönnlund et al. [43] used multi-color STED to simultaneously visualize the spatial relationships between VEGF, PF-4, fibrinogen, and actin in platelets (Fig. 3D). The abnormal localization of these angiogenesis-regulating proteins may contribute to the dysregulated blood vessel growth that drives tumor progression. Identifying these unique protein distribution signatures may contribute to the dysregulated angiogenesis that drives tumor growth and metastasis. Bergstrand et al. [84, 85] also demonstrated some significant metastasis-related proteins in platelets that co-culturing platelets with various cancer cell lines induced distinct clustering patterns include P-selectin, angiogenesis-regulating proteins and SNAREs (soluble N-ethylmaleimide factor attachment protein receptors) involved in cancer cell interactions (Fig. 3F, G).

#### Monitoring cancer therapeutics

In addition to platelets, super-resolution imaging of tumor cells themselves has uncovered novel diagnostic markers. dSTORM imaging by Nerreter and colleagues [86] detected CD19 antigens on multiple myeloma cells with superior sensitivity compared to flow cytometry (Fig. 3H). CD19 expression was detectable on a subset of myeloma cells even at levels below 100 molecules per cell. dSTORM is an effective tool in guiding therapies against heterogeneous and dynamic antigens with low density, approximately 5000 molecules per cell, such as CD20 and CD38. This finding not only identifies CD19 as a highly sensitive myeloma biomarker, but also provides a rationale for treating myeloma with CD19-targeted CAR T.

### Infectious diseases

#### Red blood cell parasite

Malaria, a widespread parasitic infection, occurs when Plasmodium sporozoites invade human red blood cells, leading to their destruction [99]. STED imaging has allowed for the observation of the complete life cycle of Plasmodium in vitro with minimal damage to the sample (Fig. 3I), offering vital insights for developing targeted malaria drugs [87]. Additionally, Mehnert et al. [24] improved the immunofluorescence staining method and cell seeding protocols for STED imaging of the malaria parasite cycle, making significant contributions to malaria research. The use of STED nanoscopy for Plasmodium imaging is significant as it reveals the nanoscale organization and dynamics of various structures and molecules critical for parasite invasion, egress, motility, and development [100].

#### Monitoring epidemic vaccine efficacy

Moreover, fluorescence nanoscopy has been used to scrutinize the impact of epidemic disease vaccine, utilizing α-granules secretin, specifically platelet factor 4 (PF4), as a biomarker for efficacy of COVID-19 vaccines. This methodology leverages the antigen complex formation between COVID-19 vaccine and the PF4, a process meticulously observed by dSTORM [88]. These insights help us understand and evaluate the molecular dynamics and cellular mechanisms underlying infectious diseases.

## Discussions and perspectives

By breaking the barriers imposed by conventional microscopy, fluorescence nanoscopy opens a window into the nanoscale intricacies of blood cells. We provide an overview of various nanoscopy technologies and their applications in studying blood cells, evaluating their developmental stages, and their challenges regarding functionality and practicality. The applications of these methodologies in mapping the distribution, organization and interactions of key proteins, understanding the dynamics of blood cell behavior, and unraveling the pathological mechanisms linked to platelet disorders, cancer, and erythrocyte parasites infection have been highlighted.

Technically, the utilization of SIM presents a remarkable opportunity for monitoring treatment responses or prognosticating blood disorders and cancer-related proteins in platelet granules. The techniques offer quantitative, fast analysis capabilities that have great potential to contribute to diagnosing diseases related to platelet granules and some distinct membrane antigens. While acknowledging the benefits of SIM, it is crucial to recognize its limitations, particularly concerning its resolution. Nonetheless, these limitations should not be seen as insurmountable barriers. Fang and his colleague [101] developed a low-toxicity, light-stable, near-infrared small-molecule fluorescent probe, named HD-BR, which has successfully enhanced SIM’s resolution to 100 nm.

It is important to acknowledge the complementary role of electron microscopy (EM) in visualizing cellular structures with unparalleled resolution. For example, cryo-EM revealed platelet granules' ultrastructure and the cytoskeletal proteins' arrangement [102, 103]. Advanced EM has been applied to study the localization of specific proteins within platelet granules and the interactions between platelets and other blood cells [104, 105]. Correlative light and electron microscopy (CLEM) technique has enabled the integration of fluorescence nanoscopy with EM, allowing for the simultaneous visualization of specific molecular targets and ultrastructural context [106]. However, EM techniques also have their limitations, such as the requirement for extensive sample preparation, the inability to image live cells, and the lack of molecular specificity. Fluorescence nanoscopy, on the other hand, allows for the dynamic imaging of live cells and the specific labeling of molecular targets. Therefore, the integration of EM and fluorescence nanoscopy will offer a promising avenue for future research in blood cell biology and disease mechanisms.

Despite the specialized analysis being time-consuming and requiring post-imaging, sensitivity and specificity affected by variable and dynamic molecules, advances in STED and SMLM imaging have revealed intricate details about protein storage and release mechanisms within platelets. These technologies have proven effective in achieving high spatial resolution and labeling accuracy, particularly in examining sub-cellular protein distribution. The nanoscale insights into the dysregulated distribution and interactions of angiogenesis-regulating proteins in platelets from cancer patients, as revealed by STED imaging [22, 43, 84, 85], provide a mechanistic basis for developing novel anti-angiogenic therapies targeting platelets. For example, drugs that normalize the spatial organization of VEGF and PF-4 in platelet α-granules could potentially inhibit tumor angiogenesis and metastasis. Similarly, the identification of CD19 as a sensitive biomarker for myeloma cells using dSTORM [86] has directly informed the use of CD19-targeted CAR T cell therapy in myeloma patients.

In terms of clinical applications, the integration of nanoscopy with AI-based image analysis, as demonstrated by Zhang et al. [83] for platelet analysis in cancer patients, holds great promise for automated, high-throughput diagnostic platforms. The development of nanoscopy-based liquid biopsy techniques, where circulating tumor cells or platelets are isolated from patient blood samples and analyzed for nanoscale protein distribution patterns, could enable minimally invasive early cancer detection and treatment monitoring. Additionally, the ability of STED nanoscopy to visualize the detailed subcellular structures of Plasmodium-infected erythrocytes [24, 87] could guide the design of targeted antimalarial drugs that specifically disrupt parasite-host interactions.

However, the diagnostic application of nanoscopy in blood cells and platelets is accompanied by inherent challenges. To optimize the performance and accuracy of super-resolution imaging, several avenues can be explored: (i) labelling improvements: one can employ genetically encoded fluorescent proteins or nanobodies. These entities bind with remarkable specificity, enhancing the labeling of cytoskeletal proteins [45, 107]; (ii) speed and temporal resolution: faster cameras, customized illumination patterns, and compressed sensing techniques could increase the speed and temporal resolution of the imaging process [108, 109]; (iii) data interpretation and accuracy: advanced algorithms and models [110] that can account for noise, artifacts, and biological variability can be used to ensuring precise data interpretation. Furthermore, integrating novel fluorescent probes, optimizing imaging parameters, creating new algorithms, and involving machine learning [111] present other promising directions to address the existing challenges.

Looking ahead, we foresee an increase in studies focused on red blood cell parasites and aging-associated proteins. As an ideal vehicle for nano-drug delivery, nanoscopy may probe the interaction between nanocarriers and cell membranes in the red blood cell hitchhiking process. Additionally, we suggest paying attention to the translocation of lipid rafts and tetraspanins on white blood cell membrane proteins, which are crucial for researching cellular functions. Except for membrane proteins, cytokines secreted by white blood cells (such as IL-1, IL-6) also manage and regulate many immune responses and inflammation reactions, however, it is a new field that can be further explored by using nanoscopy, which will contribute to related diseases monitoring and prognosis. We have underscored the significance of investigating abnormalities in platelet-specific cytoskeleton and granules proteins, as numerous studies suggest their potential as indicators for early detection of cancer, platelet disorders, as well as virus infection [97, 98, 112, 113].

Research might also delve into the functional roles of different protein layers, their regulation by mechanical and biochemical signals, and the dynamics of focal adhesion assembly and remodeling, crucial in cell migration and mechano-transduction. The nanoscale organization of focal adhesion proteins, as revealed by iPALM [73], could guide the development of drugs targeting specific focal adhesion components to inhibit cancer cell migration and metastasis. Further studies on Focal Adhesion Kinase (FAK) using super-resolution microscopy could reveal its role in cancer invasion and metastasis, opening avenues for new therapeutic strategies targeting FAK or its interacting partners in cancer cells. Super-resolution imaging may also be applied to pinpoint the molecular conformation change, such as integrin αIIbβ3 maturation, from resting to intermediate to activated conformation states, upon platelet activation during thrombosis (Fig. 1A).

At the technical level, fluorescence nanoscopy can be enhanced by integration with artificial intelligence (AI). This combination can automate processes such as amplifying fluorescence signals, segmenting and recognizing cells or organelles, and classifying them. A long-term objective for the use of nanoscopy in diagnosis is to evaluate patients’ long-term outcomes and quality of life. Identifying factors that predict survival and toxicity could offer valuable insights for enhancing the effectiveness of current therapeutic methods. Another future direction will be enabling multimodality imaging capacity using functional probes and nanoparticles [114, 115], which could report the intracellular biophysical conditions, such as in situ temperature, pH, membrane potential, etc. which are related to the healthy condition of cells, tissues or organs.

In summary, despite the technical challenges faced by current nanoscopy techniques, their potential to revolutionize medical diagnostics and disease research is undeniable. The super-resolution microscopy studies discussed in this review, along with the complementary insights provided by EM techniques, have not only advanced our understanding of the nanoscale architecture and molecular interactions in blood cells but also provided a framework for translating these insights into clinical applications. From guiding the development of targeted therapies for platelet disorders and cancer to enabling AI-powered diagnostic platforms, the nanoscale imaging of blood cells is poised to transform the way we diagnose and treat hematological disorders. We advocate for continued research and technological advancement in this field, with the aspiration that these innovative blood cell ultra-structure imaging modalities will contribute significantly to addressing clinical challenges disease diagnosis, monitoring and beyond.

## Data Availability

Not applicable.
